# Parity and NIS Expression in Atypical Cells of Triple-Negative Breast Cancer, and Prognosis

**DOI:** 10.3390/ijms26209947

**Published:** 2025-10-13

**Authors:** Grigory Demyashkin, Eugenia Kogan, Tatiana Demura, Anastasia Guzik, Dmitriy Belokopytov, Maxim Batov, Vladimir Shchekin, Irina Bicherova, Petr Shegai, Andrei Kaprin

**Affiliations:** 1 Department of Digital Oncomorphology, National Medical Research Centre of Radiology, 2nd Botkinsky Pass., 3, 125284 Moscow, Russia; doctor.guzik@mail.ru (A.G.); beldimbur@gmail.com (D.B.); md.batov@gmail.com (M.B.); dr.shchekin@mail.ru (V.S.); dr.shegai@mail.ru (P.S.); kaprin@mail.ru (A.K.); 2 Laboratory of Histology and Immunohistochemistry, Institute of Translational Medicine and Biotechnology, I.M. Sechenov First Moscow State Medical University (Sechenov University), Trubetskaya St., 8/2, 119048 Moscow, Russia; 3 Institute of Clinical Morphology and Digital Pathology, I.M. Sechenov First Moscow State Medical University (Sechenov University), Trubetskaya St., 8/2, 119048 Moscow, Russia; kogan_e_a@staff.sechenov.ru (E.K.); demura_t_a@staff.sechenov.ru (T.D.); 4 Research and Educational Resource Center for Immunophenotyping, Digital Spatial Profiling and Ultrastructural Analysis Innovative Technologies, Peoples’ Friendship University of Russia (RUDN University), Miklukho-Maklaya St., 6, 117198 Moscow, Russia; 5 Department of Morphology, Pirogov Russian National Research Medical University (Pirogov University), Ostrovityanova St., 1, 117513 Moscow, Russia; raddp8@yandex.ru; 6 Department of Urology and Operative Nephrology, Peoples’ Friendship University of Russia (RUDN University), Miklukho-Maklaya St., 6, 117198 Moscow, Russia

**Keywords:** triple-negative breast cancer, sodium/iodide symporter (NIS), breast cancer, pregnancy

## Abstract

Breast cancer is one of the most common malignancies worldwide, affecting 2.3 million and causing 670,000 deaths in women annually. However, data indicate that the risk of developing breast cancer decreases with pregnancy at a young age, and each subsequent pregnancy further reduces the risk by approximately 10%. One of the characteristics inherent in both the mammary gland epithelium in pregnant women and luminal epithelial adenocarcinomas is the increased expression of NIS—the sodium/iodide symporter, whose defective cytoplasmic forms possess pro-oncogenic properties. Therefore, the analysis of the degree of influence of pregnancy on NIS expression in breast cancer cells is of medical interest. The aim of this study is to conduct a comparative morphological analysis of NIS expression in breast cancer cells according to the number of pregnancies of each patient. This study included 161 patients with triple-negative breast cancer who visited the P.A. Herzen Moscow Oncology Research Institute from 2020 to 2023. Immunohistochemical examination was performed using antibodies to NIS. The gravidity status of women was determined based on provided medical documentation. The degree of NIS expression was assessed using a modified Gainor scale. Statistical analysis was performed using mean and standard deviation (SD) depending on the normality of the distribution (Lilliefors test: *p* > 0.20); a *p*-value ≤ 0.05 was considered statistically significant. The degree of correlation between variables was assessed using Kendall’s tau rank correlation coefficient. A weak to moderate negative correlation (τ: −0.369) was found between the number of pregnancies and the degree of NIS expression in triple-negative breast cancer cells. In patients with triple-negative breast cancer, a weak to moderate negative correlation was found between the degree of NIS expression and gravidity status. The discovered phenomenon is likely due to the terminal differentiation of the mammary gland epithelium that occurs during pregnancy. This may potentially indicate the suppression of pro-oncogenic properties of atypical cells developed from the epithelium that has undergone terminal differentiation.

## 1. Introduction

Breast cancer (BC) affects 2.3 million women worldwide and causes 670,000 deaths annually [1]. A distinctive feature of this malignancy is that the main risk factors for BC are non-modifiable. For example, reproductive factors play a key role [2]. The fact is that one of the ontogenetic features of the luminal epithelium of the mammary glands is the variability of its proliferative status depending on the current hormonal background and presence of pregnancies in the patient’s history. Molecular biological studies have demonstrated that even after one pregnancy, the proliferative activity of the epithelium of terminal ductal lobular units decreases many times over, which significantly affects the risk of malignant transformation of these cells [3]. The reason for these changes is cellular terminal differentiation, which in the long term reduces the risk of cancer development [4]. One manifestation of terminal differentiation is a change in the degree of DNA methylation, which is physiologically necessary for the rapid reactivation of specific genes required for cellular and metabolic restructuring of the mammary gland and milk secretion. Thus, in the study by dos Santos et al., it was demonstrated that pregnancy changes the degree of genome methylation due to the activity of the transcription factor STAT5a.

In this case, it is of particular interest to study changes in the biological properties of a tumor depending on the woman’s gravidity. One of the paradoxical manifestations of changes in the functioning of the genetic apparatus of tumor cells is the abnormal expression of the sodium/iodide protein co-transporter—NIS/SLC5A5, which is normally expressed in the epithelial cells of the mammary ducts only in the late stages of pregnancy and during lactation [5]. The main function of this protein is to saturate milk with iodine to maintain and stimulate the activity of the thyroid gland of the newborn, which contributes to the adequate development of the newborn organism [6]. However, in 2000, Tazebay et al. discovered that most breast carcinomas are NIS-overexpressing [7]. Further studies have demonstrated that this is likely an indirect manifestation of genetic mutations characteristic of breast carcinomas/triple-negative breast cancer, leading to hyperactivation of the FOXA1 and PI3K/AKT/mTOR signaling pathways [8,9]. At the same time, the disorganization of the protein synthetic apparatus of tumor cells leads to excessive accumulation in the cytoplasm of pathological forms of the NIS protein, which have pro-oncogenic properties—the ability to stimulate the migration and metastasis of tumor cells; that is, NIS expression itself is a factor potentially capable of influencing the prognosis in each individual patient [10]. However, despite the availability of experimental studies on the role of NIS in tumor biology over the past 20 years, there are no studies on the practical application of NIS expression assessment, and this requires separate attention.

We hypothesize that a woman’s gravidity status affects the functioning of the genetic apparatus of cancer cells, the expression of the NIS protein being an integrative indicator of its disorganization. However, despite the crucial role of NIS in breast cancer, its expression in triple-negative breast cancer (TNBC) depending on age and its relationship with gravidity status are poorly understood. To exclude the influence of background hormonal factors that can significantly affect the biology of the tumor, the study was carried out among a cohort of patients with triple-negative breast cancer. Thus, we conducted a comparative complex clinical and morphological analysis of sodium/iodide symporter (NIS) expression in triple-negative breast cancer depending on the number of pregnancies. In this case, we focused on the prognostic value of NIS expression using various methods, primarily immunohistochemical research.

### Research Objective

**Purpose:** To investigate the degree of correlation between NIS expression in atypical cells of triple-negative breast cancer and the gravidity status of women.

## 2. Results

### 2.1. Clinical Data

The study included 161 patients with a median age of 53.9 years. The distribution of patients by disease stage according to the TNM classification is shown in Table 1. The majority of patients at the time of presentation had stage IIA (54.66%), and were referred for treatment during a routine annual examination and mammography. The physiological and functional status corresponded to 0–1 points on the ECOG scale for all patients. The predominant side of the lesion was the right (*n* = 113; 70.1%), the left side was observed in 40 patients (24.8%), and 8 had bilateral lesions (4.9%). In outpatient settings, all women underwent a biopsy of the tumor focus under sonography control, followed by morphological examination. The main clinicopathologic features of the study cohort are outlined in Table 2.

### 2.2. Genetic Testing

Genetic testing of peripheral blood samples using real-time polymerase chain reaction (DTPrime4; DNA-technology) in patients (*n* = 161) revealed no mutations in the *BRCA1*, *BRCA2*, and *CHEK2* genes.

### 2.3. Morphological Examination

**Figure 1:**
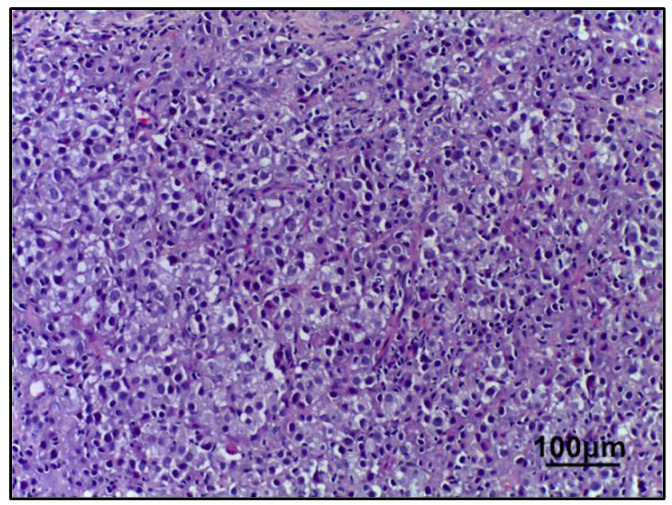
Invasive breast carcinoma (no special type). Hematoxylin & eosin staining (200× magnification).

In breast tissue samples/pre-NAC biopsies from all patients (*n* = 161), a morphological picture of invasive, non-specific type carcinoma (G3 according to the Nottingham grading system in the Bloom–Richardson modification), was found. This included microfragments of the mammary gland with pathologically altered tissue (tissue and cellular atypia of the parenchyma) with accumulation of pleomorphic atypical cells (>300) forming solid structures. The cytoplasm of these cells was in the form of a thin rim; their nuclei, with signs of polymorphism, are located eccentrically and are round in shape with an “eaten” karyolemma. They have a stromal component with a weak desmoplastic reaction (Figure 1).

**Figure 2:**
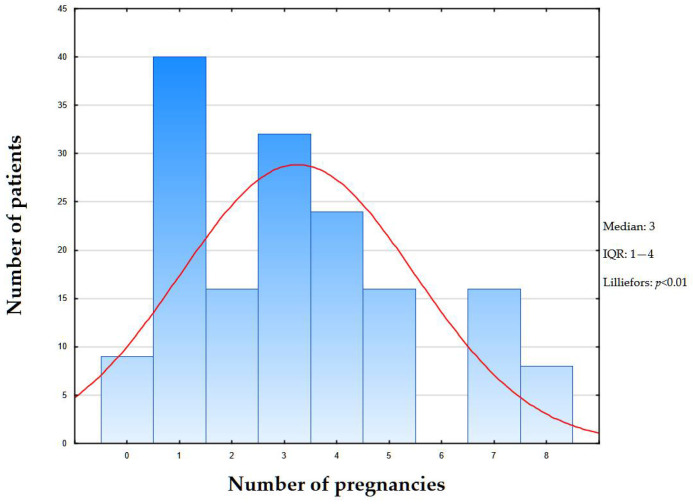
Distribution of pregnancy counts in study cohort. Bars represent number of patients for each pregnancy count (x-axis: number of pregnancies; y-axis: number of patients). Red line—expected normal.

Subsequently, based on the immunophenotyping performed, all patients were diagnosed with the triple-negative surrogate molecular genetic subtype of breast cancer. The main focus of important theoretical and practical significance was analysis of the correlation between the degree of NIS expression in atypical mammary gland cells and the number of pregnancies in the patient’s history. In a retrospective analysis of the gravidity status of patients in the cohort (*n* = 161) according to medical records, the median number of pregnancies was three (IQR: 1–4) (Figure 2), while the number of patients without pregnancies in the history was nine (5.5%).

**Figure 3:**
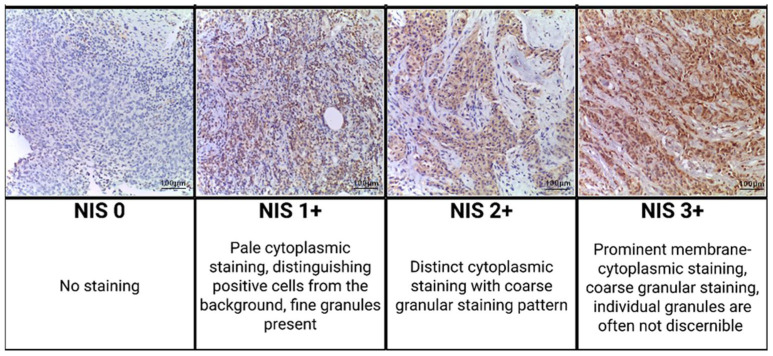
Representative immunohistochemical staining results of NIS in the invasive carcinoma component of triple-negative breast cancer; staining intensity criteria (200× magnification).

Immunohistochemical examination with antibodies to NIS revealed a positive cytoplasmic reaction in atypical cells of triple-negative breast cancer, but the degree of its severity varied. In all NIS-positive cases, staining was observed in >95% of cells; uniform distribution (Figure 3).

**Figure 4:**
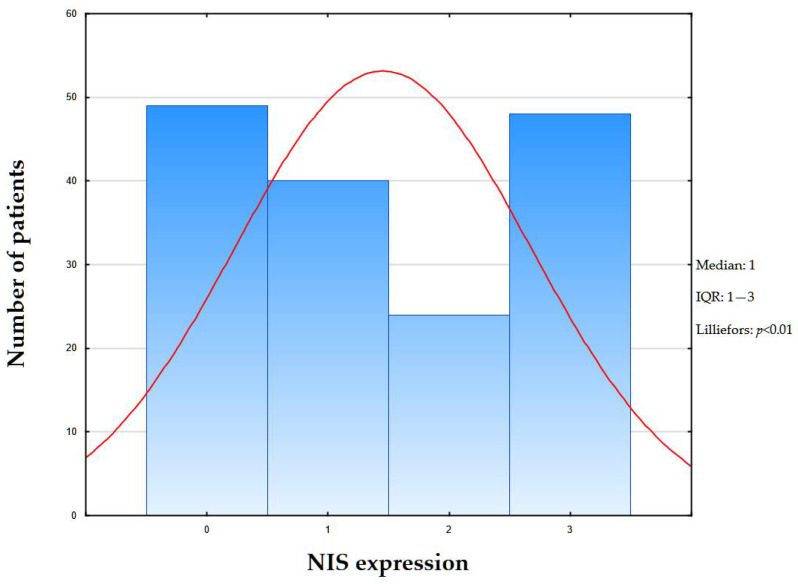
Distribution of NIS staining intensity in study cohort. Bars represent number of patients for each pregnancy count (x-axis: number of pregnancies; y-axis: number of patients). Red line—expected normal.

NIS-immunopositive reactions were found in 112 (69.5%) patients, while NIS 1+ was observed in 40 (35.7%) cases; NIS 2+ in 24 (21.4%) cases; and NIS 3+ in 48 (42.8%) cases. Triple-negative breast cancer was NIS-negative in 49 (30.4%) women, 9 of whom had no history of pregnancy. The median expression level was 1 (IQR: 1–3) (Figure 4).

After obtaining data from immunohistochemical examination with antibodies to NIS and comparing it with the gravidity status of patients, a statistical evaluation of the degree of correlation was performed using the Kendall tau coefficient to analyze the influence of the number of pregnancies on the degree of NIS expression (Table 3).

**Figure 5:**
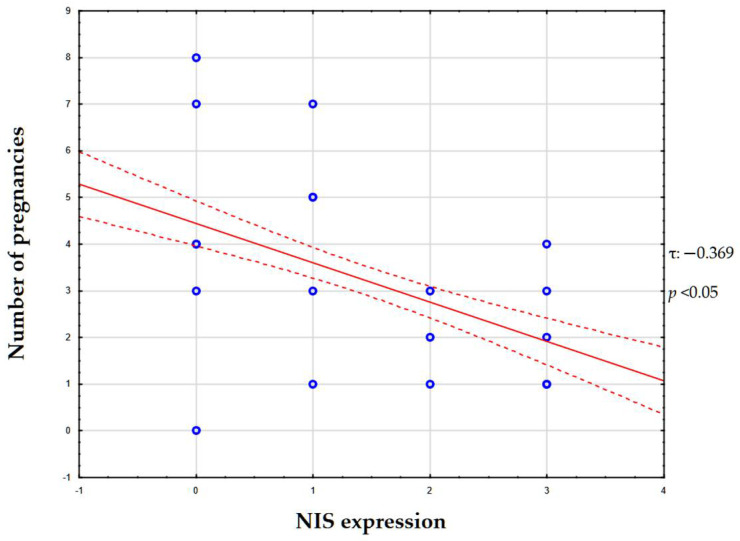
Correlation graph between NIS expression and number of pregnancies. Blue circles represent individual data points for each patient (x-axis: NIS expression score from 0 to 3+; y-axis: number of pregnancies). Solid red line indicates regression line. Dashed red lines represent 95% confidence interval around regression line.

According to the results of statistical analysis of the correlation between NIS expression level and the woman’s gravidity status, the Kendall tau coefficient was −0.369 (*p* < 0.05), which corresponds to a weak–moderate negative correlation (Figure 5).

Summarizing the above, we conducted a study of triple-negative breast cancer samples (*n* = 161), confirmed by histological and immunohistochemical examination (ER−/0; PR−/0; HER2/neu −/0), with antibodies to NIS and subsequent comparison with the gravidity status of patients.

## 3. Discussion

This study is devoted to the analysis of the correlation between the expression of the sodium-iodide symporter in atypical cells of triple-negative breast cancer and pregnancy. According to meta-analysis and patent search, a similar study has not been conducted.

According to the results of our study, it was found that in patients, 69.5% of tumors (*n* = 112) were NIS-positive, of which NIS 1+ was 35.7% of cases (*n* = 40), NIS 2+ was 21.4% (*n* = 24) and NIS 3+ was 42.8% (*n* = 48). In all NIS-positive cases, the percentage of NIS-positive tumor cells was above 95%. NIS-negative cases were 30.4% (*n* = 49). When studying the relationship between the degree of expression, it was found that the degree of NIS expression has an inverse correlation with the gravidity status of the woman; i.e., a greater number of pregnancies in the history corresponds to a lower NIS expression. Considering that NIS overexpression in atypical cells of breast carcinomas is associated with increased genetic instability and excessive activity of pro-oncogenic signaling pathways, and that intracellular accumulation of abnormal forms of NIS contributes to the migration and metastasis of tumor cells, it is more likely that the changes in the genetic and transcriptional apparatus of cells acquired by the luminal epithelium during pregnancy potentially contribute to the suppression of the malignant properties of tumor cells of triple-negative breast cancer.

Due to the highly complex influence of pregnancy on biological characteristics of the mammary gland epithelium, in this article we do not aim to encompass them all, but will focus only on certain facts and phenomena that could potentially explain our results.

First of all, it should be said that most studies, such as those conducted by Feigman et al., agree that the changes acquired by mammary gland epithelial cells during pregnancy lie in the plane of epigenetic changes. Thus, the population of epithelial cells before pregnancy and the post-gestational population differ radically at the epigenetic level—pregnancy increases the number of active enhancers by 12 times, from 5 thousand to 60 thousand. This increase in the activity of the genetic apparatus paradoxically provides an anti-oncogenic effect by enhancing the control of proliferative activity, including by enhancing the expression of p53 [11].

In addition, the extracellular matrix formed during pregnancy in the mammary gland stroma differs in the architecture of collagen fibers, the properties of which suppress pro-oncogenic signaling pathways and reduce both the risk of malignancy and the rate of proliferation, migration, and metastasis of cancer cells [12].

In parallel, increased p53 expression allows the epithelial cell to avoid malignancy and enter a senescent-like, relatively stable state. In addition, it is well known that p53, among other things, suppresses NIS expression [13]. However, it is known that triple-negative breast cancer is primarily associated with mutations in the *TP53* gene, which does not allow us to speak, within the framework of this study, as the only or key mechanism affecting the biology of tumor cells and NIS expression, in particular. Therefore, we suggest the presence in TNBC of other regulators of NIS expression, also subject to the patient’s gravidity status—one of which is *FOXA1*, which is confirmed by a lot of experimental data. First of all, there is evidence suggesting that *FOXA1* expression and methylation vary depending on a woman’s gravidity status, and a greater number of pregnancies is associated with decreased *FOXA1* expression in hormone-negative breast tumors [14].

For example, back in 2000, it became known that treatment of breast tumor cells with retinoic acid leads to increased NIS expression, and one of the properties of retinoic acid, discovered in the field of developmental biology, is the enhancement of *FOXA1* expression [15,16]. However, the most reliable and detailed data on this issue were provided by Rathod et al., who confirmed the key role of *FOXA1* as the main transcriptional regulator of NIS in atypical breast cancer cells [8].

**Figure 6:**
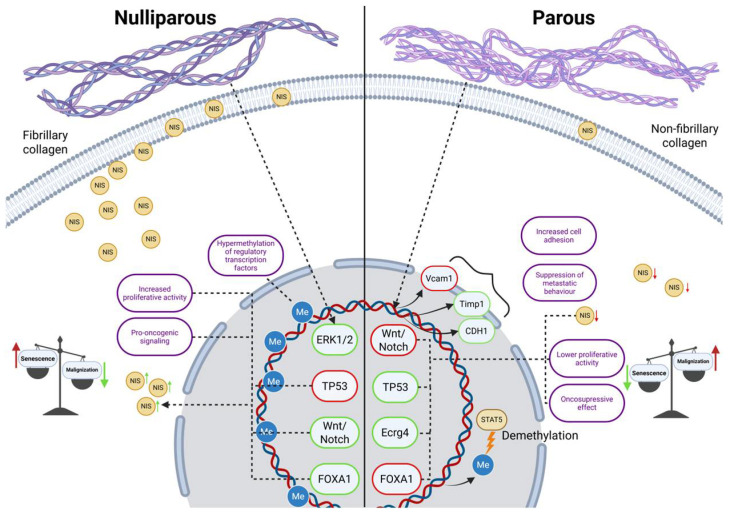
Effect of parity on tumor cell biology. Green markers represent enhancive effect; red markers represent suppressive effect.

Based on the above data, it can be summarized that in NIS-overexpressing tumors, increased FOXA1 activity is also observed, which differs relative to earlier studies in this area [17,18]. However, the 2018 work by Séverine Guiu et al., demonstrated the existence of FOXA1-positive TNBC, associated with a more negative clinical prognosis [19]. The existence of separate subtypes within TNBC is not surprising, considering the fact that triple-negative breast cancer is an umbrella term that unites extremely heterogeneous subtypes of tumors, and therefore requires an individual approach to the selection of therapy within the framework of personalized medicine [20]. The implication of NIS and pregnancy in tumor biology, based on literature data, are summarized in Figure 6.

**Figure 7:**
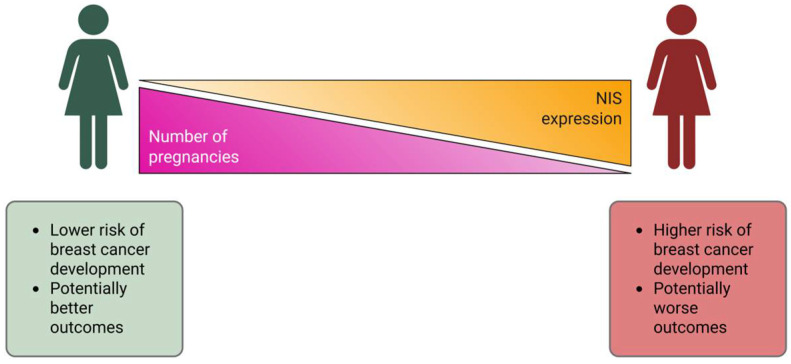
Correlation between NIS expression and parity status, clinical implications.

We also consider it important to clarify that the sample of patients we collected was quite multinational and cases of patients with multiple pregnancies (three or more) were characteristic mainly of the Caucasus region. Thus, the study conducted, devoted to the assessment of the degree of NIS expression in atypical cells of triple-negative breast cancer, has important fundamental significance, namely the influence of the number of pregnancies on the instability of the genetic apparatus of tumor cells (Figure 7).

In addition, NIS can be considered as a potential diagnostic marker in triple-negative breast cancer for determining organ affiliation, including for the detection of the primary focus. Also, it is possible that the NIS/pregnancy correlation we found may potentially indicate a positive effect of previous pregnancies on the clinical course of breast cancer. Identification of the relationship between NIS expression and FOXA1 activity, as well as its clinical significance, will be the goal of our further research in this area.

Limitations of the study: (1) Retrospective design—reliance on medical records for pregnancy history can introduce recall bias or inaccuracies. (2) Sample size—while *n* = 161 is respectable, the subgroup of nulliparous women is very small (*n* = 9), which may limit the robustness of comparisons involving this group.

## 4. Materials and Methods

### 4.1. Patients and Clinico-Morphological Data

**Figure 8:**
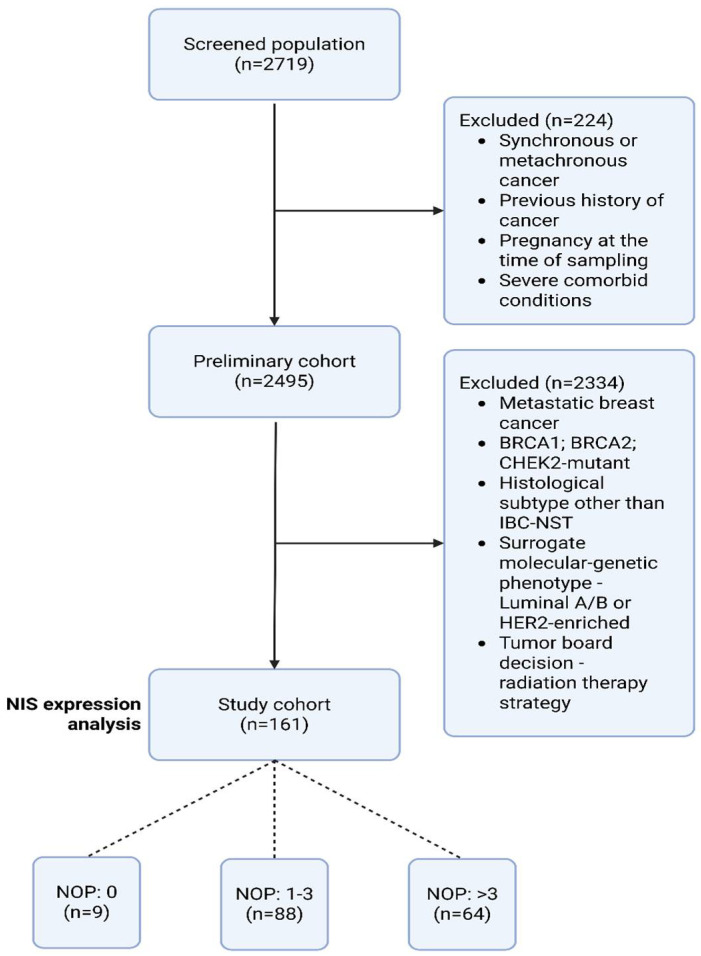
Study flowchart. Abbreviations: NOP—number of pregnancies.

In this retrospective study, the medical records of patients (*n* = 2719) with biopsy, neoadjuvant chemotherapy (NAC), and mastectomy, treated from 2020 to 2023 at the Department of Oncology and Reconstructive-Plastic Surgery of the Mammary Gland and Skin of the P.A. Herzen Institution were analyzed; age—25–75 years (median—53.9 years), with a confirmed diagnosis of breast cancer stage ≥T1a, histological subtype—invasive carcinoma of no special type (ICD-O: 8500/3), triple-negative surrogate molecular genetic subtype (ER 0/−; PR 0/−; HER2/negative). Total diagnosis—triple-negative breast cancer (*n* = 161). Inclusion and exclusion criteria are presented in Table 4. The gravidity status of women was determined based on the anamnesis and medical documentation, while only pregnancies that reached the 3rd trimester were taken into account (Figure 8).

### 4.2. Real-Time Polymerase Chain Reaction BRCA1/2 and CHEK2 Mutation Analysis

Venous blood samples were obtained from all patients participating in the study and subsequently analyzed for *BRCA1/2* gene mutations using the real-time polymerase chain reaction method (DTPrime4; DNA-technology). The mutations investigated in the *BRCA1* gene: 5382insC (insertion of C at position 5382); 4153delA (deletion of A at position 4153); 300T/G (substitution of T to G at position 300); 3819delGTAAA (deletion of GTAAA at position 3819); 2080delA (deletion of A at position 2080); 185delAG (deletion of AG at position 185); and 3875delGTCT (deletion of GTCT at position 3875). BRCA2 gene: 6174delT (deletion of T at position 6174). A germline mutation analysis of the CHEK2 gene associated with breast and ovarian cancer was also performed: the mutations investigated were 1100delC (deletion of C at position 1100); IVS2+1G>A (substitution of G to A at position +1 of intron 2 splicing site); and 470T>C (substitution of T to C at position 470).

### 4.3. Morphological Block

Breast tissue fragments—pre-NAC biopsy (*n* = 161), were fixed in buffered formalin solution, processed automatically, embedded in paraffin blocks (FFPE), and sectioned into serial slices with a thickness of 2 μm. The sections were then deparaffinized, dehydrated, and stained with hematoxylin and eosin. The evaluation of the specimens was conducted according to standard histological criteria.

Immunohistochemical staining on 161 TNBC pre-NAC biopsy was performed. The triple-negative subtype was diagnosed based on the College of American Pathology guidelines for Breast Biomarker Reporting March 2023, using the Allred score and standard HER2/neu assessment. The following primary antibodies were used: monoclonal antibodies against the sodium/iodide symporter (NIS/SL5A5; Affinity Biosciences Cat# DF2242, RRID: AB_2839473). For secondary antibody detection, a universal two-component HiDef Detection™ HRP Polymer system (Cell Marque, Rocklin, CA, USA) was used, including anti-IgG mouse/rabbit antibodies, horseradish peroxidase (HRP), and DAB substrate. Cell nuclei were counterstained with Mayer’s hematoxylin. We counted the percentage of positive tumor cells from 0 to 100%. The tissue staining intensity was graded using the modified Gainor’s semi-quantitative method from 0 to 3+, as follows: 0 points—staining absent; 1 point—pale cytoplasmic staining, distinguishing positive cells from the background, fine granules present; 2 points—distinct cytoplasmic staining with coarse granular staining pattern; 3 points—prominent membrane-cytoplasmic staining, coarse granular staining, individual granules are often not discernible [21].

The positive control tissue sample was thyroid with Grave’s disease [22]. Microscopic analyses were conducted utilizing a video microscopy system comprising a Leica DM3000 microscope (Leica Microsystems, Wetzlar, Germany), a Leica ICC50 HD camera (Leica Microsystems Gmbh, Wetzlar, Germany), and a Platrun LG computer (LG Electronics Inc., Seoul, South Korea). Morphometric data were acquired through Leica Application Suite (LAS) Version 4.9.0 image processing and analysis software. Quantification was assessed as the number of positive cells per 1 mm^2^ using Qupath software (ver. 0.5.1).

### 4.4. Statistical Analysis

Statistical analysis of the sample was performed using Statistica 13.5.0.17 software (TIBCO Software Inc., Palo Alto, CA, USA). The description of quantitative data included the determination of the median and interquartile range (IQR; 25–75 percentile) or mean and standard deviation (SD) depending on the normality of the distribution (Lilliefors test: *p* > 0.20); a *p*-value ≤ 0.05 was considered statistically significant. The degree of correlation between variables was assessed using Kendall’s tau rank correlation coefficient.

## 5. Conclusions

In patients with triple-negative breast cancer, a weak to moderate negative correlation was found between the degree of NIS expression and gravidity status. The discovered phenomenon is likely due to the terminal differentiation of the mammary gland epithelium that occurs during pregnancy. This may potentially indicate the suppression of pro-oncogenic properties of atypical cells of epithelial origin that have undergone terminal differentiation. Furthermore, in our study we found no correlation between the degree of NIS expression and patient age, tumor size, number of affected lymph nodes, or presence of lymphovascular invasion. Therefore, our main hypothesis requires further confirmation by conducting large prospective studies using molecular biological and molecular genetic methods.

## Figures and Tables

**Table 1:** Distribution of patients by TNM disease stage.

TNM Stage	Number of Patients (%)
Stage IA	8 (4.97%)
Stage IB	1 (0.62%)
Stage IIA	88 (54.66%)
Stage IIB	16 (9.94%)
Stage IIIA	8 (4.97%)
Stage IIIB	16 (9.94%)
Stage IIIC	24 (14.9%)
Stage IV	0
Total: 161 (100%)	

**Table 2:** Clinicopathologic features of 161 patients with surgically resected TNBC.

Variables	TNBC Samples (Total *n* = 161)
Age (years)	
Mean ± SD	53.6 ± 11.97
Range	25–75
Sex (*n*, %)	
Male	0
Female	161 (100%)
Histologic type (*n*, %)	
IBC-NST	161 (100%)
Tumor size (cm)	
Median [IQR]	2.5 [2.2–3.5]
Range	0.7–5.6

**Table 3:** Clinicopathologic features of 161 patients with surgically resected TNBC. Correlation with NIS expression.

Variables	TNBC Samples (Total *n* = 161)				
	NIS Expression				
	NIS 0	NIS 1+	NIS 2+	NIS 3+	Kendall Tau Correlation *p*-Value
Age (years) (n, %)					
<45	17 (10.56%)	6 (3.73%)	0	16 (9.94%)	0.073
≥45	32 (19.88%)	34 (21.12%)	24 (14.91%)	32 (19.88%)	
**cT stage (*n*, %)**					
1	1 (0.62%)	0	8 (4.97%)	0	0.507
2	40 (24.84%)	40 (24.84%)	8 (4.97%)	40 (24.84%)	
3	8 (4.97%)	0	0	0	
4	0	0	8 (4.97%)	8 (4.97%)	
**cN stage (*n*, %)**					
0	40 (24.84%)	16 (9.94%)	16 (9.94%)	40 (24.84%)	0.240
1	0	8 (4.97%)	8 (4.97%)	0	
2	0	8 (4.97%)	0	0	
3	9 (5.59%)	8 (4.97%)	0	8 (4.97%)	
**Lymphovascular invasion (*n*, %)**					
Not identified	40 (24.84%)	16 (9.94%)	16 (9.94%)	40 (24.84%)	0.390
Present	9 (5.59%)	24 (14.9%)	8 (4.97%)	8 (4.97%)	
**Number of pregnancies (*n*, %)**					
0	9 (5.5%)	0	0	0	<0.05
1–3	8 (4.97%)	16 (9.94%)	24 (14.9%)	40 (24.8%)	
3+	32 (19.87%)	24 (14.9%)	0	8 (4.97%)	

**Table 4:** Inclusion and exclusion criteria.

Inclusion Criteria	Exclusion Criteria
✓ Female sex ✓ Age ≥ 18 years ✓ Newly diagnosed breast cancer (≥T1a stage) ✓ Histological subtype—invasive carcinoma of no special type (ICD-O: 8500/3) ✓ Surrogate molecular genetic subtype—triple negative (*n* = 161)	✓ Pregnancy at the time of sampling ✓ Severe comorbid conditions (CHF stage III–IV according to NYHA, sepsis, etc.) ✓ Synchronous or metachronous cancer ✓ History of previous malignant diseases ✓ BRCA1; BRCA2; CHEK2 mutations

## Data Availability

The data presented in this study are available on request from the corresponding author due to privacy restrictions, but include an anonymized dataset of clinicopathological parameters and NIS expression scores for the 161 patients included in the study.

## Abbreviation

BC	Breast Cancer
BRCA1	Breast Cancer Gene 1
BRCA2	Breast Cancer Gene 2
CHEK2	Checkpoint Kinase 2
CHF	Congestive Heart Failure
DAB	Diaminobenzidine (substrate for immunohistochemical detection)
ECOG	Eastern Cooperative Oncology Group (performance status scale)
ER	Estrogen Receptor
FOXA1	Forkhead Box A1 (transcription factor)
HER2/neu	Human Epidermal Growth Factor Receptor 2
HRP	Horseradish Peroxidase
IBC-NST	Invasive Breast Carcinoma of No Special Type
ICD-O	International Classification of Diseases for Oncology
ICMJE	International Committee of Medical Journal Editors
IQR	Interquartile Range
NIS	Sodium/Iodide Symporter (syn. SLC5A5 protein)
NOP	Number of Pregnancies
NYHA	New York Heart Association (heart failure classification)
PI3K/AKT/mTOR	Phosphoinositide 3-Kinase/Protein Kinase B/Mammalian Target of Rapamycin (signaling pathway)
PR	Progesterone Receptor
SD	Standard Deviation
SLC5A5	Solute Carrier Family 5 Member 5 (gene for NIS)
STAT5a	Signal Transducer and Activator of Transcription 5a
TNBC	Triple-Negative Breast Cancer
TNM	Tumor Node Metastasis (cancer staging system)
TP53	Tumor Protein 53 (gene for p53)

## References

[1] Kim J., Harper A., McCormack V., Sung H., Houssami N., Morgan E., Mutebi M., Garvey G., Soerjomataram I., Fidler-Benaoudia M.M. (2025). Global patterns and trends in breast cancer incidence and mortality across 185 countries. Nat. Med..

[2] Sprague B.L., Trentham-Dietz A., Egan K.M., Titus-Ernstoff L., Hampton J.M., Newcomb P.A. (2008). Proportion of invasive breast cancer attributable to risk factors modifiable after menopause. Am. J. Epidemiol..

[3] Slepicka P.F., Cyrill S.L., Dos Santos C.O. (2019). Pregnancy and Breast Cancer: Pathways to Understand Risk and Prevention. Trends Mol. Med..

[4] Ali S., Hamam D., Liu X., Lebrun J.J. (2019). Terminal differentiation and anti-tumorigenic effects of prolactin in breast cancer. Front. Endocrinol..

[5] Ravera S., Reyna-Neyra A., Ferrandino G., Amzel L.M., Carrasco N. (2017). The Sodium/Iodide Symporter (NIS): Molecular Physiology and Preclinical and Clinical Applications. Annu. Rev. Physiol..

[6] Beyer S.J., Jimenez R.E., Shapiro C.L., Cho J.Y., Jhiang S.M. (2009). Do cell surface trafficking impairments account for variable cell surface sodium iodide symporter levels in breast cancer?. Breast Cancer Res. Treat..

[7] Tazebay U.H., Wapnir I.L., Levy O., Dohan O., Zuckier L.S., Zhao Q.H., Deng H.F., Amenta P.S., Fineberg S., Pestell R.G. (2000). The mammary gland iodide transporter is expressed during lactation and in breast cancer. Nat. Med..

[8] Rathod M., Kelkar M., Valvi S., Salve G., De A. (2020). FOXA1 Regulation Turns Benzamide HDACi Treatment Effect-Specific in BC, Promoting NIS Gene-Mediated Targeted Radioiodine Therapy. Mol. Ther. Oncolytics.

[9] Lacoste C., Hervé J., Nader M.B., Dos Santos A., Moniaux N., Valogne Y., Montjean R., Dorseuil O., Samuel D., Cassio D. (2012). Iodide Transporter NIS Regulates Cancer Cell Motility and Invasiveness by Interacting with the Rho Guanine Nucleotide Exchange Factor LARG. Cancer Res..

[10] Micali S., Bulotta S., Puppin C., Territo A., Navarra M., Bianchi G., Damante G., Filetti S., Russo D. (2014). Sodium iodide symporter (NIS) in extrathyroidal malignancies: Focus on breast and urological cancer. BMC Cancer.

[11] Feigman M.J., Moss M.A., Chen C., Cyrill S.L., Ciccone M.F., Trousdell M.C., Yang S.-T., Frey W.D., Wilkinson J.E., dos Santos C.O. (2020). Pregnancy reprograms the epigenome of mammary epithelial cells and blocks the development of premalignant lesions. Nat. Commun..

[12] Maller O., Hansen K.C., Lyons T.R., Acerbi I., Weaver V.M., Prekeris R., Tan A.-C., Schedin P. (2013). Collagen architecture in pregnancy-induced protection from breast cancer. J. Cell Sci..

[13] Kelkar M.G., Thakur B., Derle A., Chatterjee S., Ray P., De A. (2017). Tumor suppressor protein p53 exerts negative transcriptional regulation on human sodium iodide symporter gene expression in breast cancer. Breast Cancer Res. Treat..

[14] Espinal A.C., Buas M.F., Wang D., Cheng D.T.-Y., Sucheston-Campbell L., Hu Q., Yan L., Payne-Ondracek R., Cortes E., Tang L. (2017). FOXA1 hypermethylation: Link between parity and ER-negative breast cancer in African American women?. Breast Cancer Res. Treat..

[15] Kogai T., Schultz J.J., Johnson L.S., Huang M., Brent G.A. (2000). Retinoic acid induces sodium/iodide symporter gene expression and radioiodide uptake in the MCF-7 breast cancer cell line. Proc. Natl. Acad. Sci. USA.

[16] Lavudi K., Nuguri S.M., Olverson Z., Dhanabalan A.K., Patnaik S., Kokkanti R.R. (2023). Targeting the retinoic acid signaling pathway as a modern precision therapy against cancers. Front. Cell Dev. Biol..

[17] Cheng T.-Y.D., Yao S., Omilian A.R., Khoury T., Buas M.F., Payne-Ondracek R., Sribenja S., Bshara W., Hong C.-C., Bandera E.V. (2020). FOXA1 Protein Expression in ER^+^ and ER^-^ Breast Cancer in Relation to Parity and Breastfeeding in Black and White Women. Cancer Epidemiol. Biomark. Prev..

[18] Chen Q.-X., Yang Y.-Z., Liang Z.-Z., Chen J.-L., Li Y.-L., Huang Z.-Y., Weng Z.-J., Zhang X.-F., Guan J.-X., Tang L.-Y. (2021). Time-varying effects of FOXA1 on breast cancer prognosis. Breast Cancer Res. Treat..

[19] Guiu S., Mollevi C., Charon-Barra C., Boissière F., Crapez E., Chartron E., Lamy P.-J., Gutowski M., Bourgier C., Romieu G. (2018). Prognostic value of androgen receptor and FOXA1 co-expression in non-metastatic triple negative breast cancer and correlation with other biomarkers. Br. J. Cancer.

[20] Derakhshan F., Reis-Filho J.S. (2022). Pathogenesis of Triple-Negative Breast Cancer. Annu. Rev. Pathol. Mech. Dis..

[21] Gainor D.L., Chute D.J., Lorenz R.R. (2015). Sodium Iodide Symporter Expression in Adenoid Cystic Carcinoma of the Head and Neck. Arch. Otolaryngol. Neck Surg..

[22] Castro M.R., Bergert E.R., Beito T.G., McIver B., Goellner J.R., Morris J.C. (1999). Development of monoclonal antibodies against the human sodium iodide symporter: Immunohistochemical characterization of this protein in thyroid cells. J. Clin. Endocrinol. Metab..

