# Correction: Demyashkin et al. Parity and NIS Expression in Atypical Cells of Triple-Negative Breast Cancer, and Prognosis. *Int. J. Mol. Sci.* 2025, *26*, 9947

**DOI:** 10.3390/ijms262311499

**Published:** 2025-11-27

**Authors:** Grigory Demyashkin, Eugenia Kogan, Tatiana Demura, Anastasia Guzik, Dmitriy Belokopytov, Maxim Batov, Vladimir Shchekin, Irina Bicherova, Petr Shegai, Andrei Kaprin

**Affiliations:** 1 Department of Digital Oncomorphology, National Medical Research Centre of Radiology, 2nd Botkinsky Pass., 3, 125284 Moscow, Russia; doctor.guzik@mail.ru (A.G.); beldimbur@gmail.com (D.B.); md.batov@gmail.com (M.B.); dr.shchekin@mail.ru (V.S.); dr.shegai@mail.ru (P.S.);; 2 Laboratory of Histology and Immunohistochemistry, Institute of Translational Medicine and Biotechnology, I.M. Sechenov First Moscow State Medical University (Sechenov University), Trubetskaya St., 8/2, 119048 Moscow, Russia; 3 Institute of Clinical Morphology and Digital Pathology, I.M. Sechenov First Moscow State Medical University (Sechenov University), Trubetskaya St., 8/2, 119048 Moscow, Russia; kogan_e_a@staff.sechenov.ru (E.K.); demura_t_a@staff.sechenov.ru (T.D.); 4 Research and Educational Resource Center for Immunophenotyping, Digital Spatial Profiling and Ultrastructural Analysis Innovative Technologies, Peoples’ Friendship University of Russia (RUDN University), Miklukho-Maklaya St., 6, 117198 Moscow, Russia; 5 Department of Morphology, Pirogov Russian National Research Medical University (Pirogov University), Ostrovityanova St., 1, 117513 Moscow, Russia; raddp8@yandex.ru; 6 Department of Urology and Operative Nephrology, Peoples’ Friendship University of Russia (RUDN University), Miklukho-Maklaya St., 6, 117198 Moscow, Russia

## Additional Affiliations

In the published publication [1], there was an error regarding the affiliations of Eugenia Kogan, Tatiana Demura, and Vladimir Shchekin. The authors inadvertently listed the incorrect departmental affiliation for three co-authors. 

The affiliation for Affiliation 1 has been refined to include the specific department: Department of Digital Oncomorphology, National Medical Research Centre of Radiology, 2nd Botkinsky Pass., 3, 125284 Moscow, Russia; doctor.guzik@mail.ru (A.G.); beldimbur@gmail.com (D.B.); md.batov@gmail.com (M.B.); dr.shchekin@mail.ru (V.S.); dr.shegai@mail.ru (P.S.); kaprin@mail.ru (A.K.)

In addition to affiliations 1, 2, 5 and 6, the updated affiliations should include the following:

Institute of Clinical Morphology and Digital Pathology, I.M. Sechenov First Moscow State Medical University (Sechenov University), Trubetskaya St., 8/2, 119048 Moscow, Russia; kogan_e_a@staff.sechenov.ru (E.K.); demura_t_a@staff.sechenov.ru (T.D.)

Research and Educational Resource Center for Immunophenotyping, Digital Spatial Profiling and Ultrastructural Analysis Innovative Technologies, Peoples’ Friendship University of Russia (RUDN University), Miklukho-Maklaya St., 6, 117198 Moscow, Russia

The authors state that the scientific conclusions are unaffected. This correction was approved by the Academic Editor. The original publication has also been updated.
